# Supplementary material for: Definition of IgG Subclass-Specific Glycopatterns in Idiopathic Membranous Nephropathy: Aberrant IgG Glycoforms in Blood
Source: Int J Mol Sci. 2022 Apr 23;23(9):4664. doi: 10.3390/ijms23094664 (PMC9101794; doi:10.3390/ijms23094664)
Supplement: Supplementary file 1 [file ijms-23-04664-s001.zip › ijms-1639421-supplementary.pdf]

International Journal of *Molecular Sciences*

MDPI

Definition of IgG Subclass-Specific Glycopatterns in Idiopathic Membranous Nephropathy: Aberrant IgG Glycoforms in Blood

Clizia Chinello [1,*], Noortje de Haan [2,3], Giulia Capitoli [4], Barbara Trezzi [5], Antonella Radice [6], Lisa Pagani [1], Lucrezia Criscuolo [1], Stefano Signorini [7], Stefania Galimberti [4], Renato Alberto Sinico [5], Manfred Wuhrer [2] and Fulvio Magni [1]

SUPPLEMENTARY MATERIAL

PLATE 1

	1	2	3	4	5	6	7	8	9	10	11	12
A	F	F	F	M	F	M	F	M	F	F	0	F
B	M	F	M	M	F	F	F	0	F	M	F	M
C	M	M	M	F	M	M	0	M	M	M	M	F
D	F	M	M	M	0	M	M	F	M	M	F	M
E	0	M	F	0	M	F	F	M	M	M	M	F
F	M	M	M	F	M	F	F	M	M	0	0	M
G	M	M	M	M	F	M	M	M	F	M	M	M
H	0	F	M	F	M	F	M	F	M	M	M	M

	1	2	3	4	5	6	7	8	9	10	11	12
A	47	83	40	35	41	81	67	51	17	77	0	78
B	78	68	29	67	51	51	53	0	70	38	40	51
C	56	58	32	50	47	58	0	56	73	74	63	83
D	39	64	35	36	0	65	53	45	28	28	39	38
E	0	74	43	0	70	19	23	37	66	77	80	78
F	38	66	40	39	66	41	47	86	42	0	0	67
G	37	48	70	53	70	51	56	41	54	58	56	71
H	0	23	43	72	51	73	71	73	60	57	67	69

PLATE 2

	1	2	3	4	5	6	7	8	9	10	11	12
A	F	M	F	M	F	0	M	0	F	M	F	F
B	M	0	F	M	M	F	M	F	M	M	M	M
C	F	M	M	M	F	F	0	M	M	F	M	F
D	M	M	F	M	M	0	M	F	F	M	M	M
E	M	M	0	M	M	M	F	M	M	M	M	F
F	F	M	M	M	M	F	M	F	M	F	0	M
G	M	F	F	M	M	M	F	F	M	M	F	0
H	M	F	M	M	M	M	F	0	M	F	M	M

	1	2	3	4	5	6	7	8	9	10	11	12
A	39	87	49	85	81	0	42	0	70	70	72	49
B	44	0	19	56	71	44	39	40	43	47	65	55
C	39	48	74	31	73	73	0	34	70	60	76	74
D	41	67	60	46	58	0	73	44	75	64	78	47
E	35	69	0	47	76	75	61	48	67	76	71	38
F	71	54	34	44	75	71	51	44	78	82	0	39
G	47	49	82	39	37	51	86	23	56	58	66	0
H	75	83	68	43	51	44	74	0	68	74	38	30

M Males
F Females
0 Standards /Blanks

Young ← AGE RANGE → Old

	Average age	Average sex (F/M)
**Plate 1**	54.67	0.368
**Plate 2**	57.70	0.356
**Total**	56.18	0.362

F = Female; M = Male; a value of zero indicates standards or blanks.

Figure S1. Age (years) and sex randomization of the cohort on sample plates.

Table S1. 56 extracted features.

Composition	Depiction	[M+3H]$^{3+}$ when extracted		
		IgG1	IgG2	IgG4
H3N4	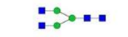	830.00	819.33	
H3N4F1	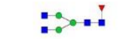	878.68	868.02	873.35
H4N4	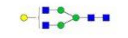	884.02	873.35	878.69
H3N5	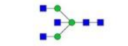	897.69		
H4N4F1	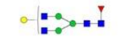	932.70	922.04	927.37
H5N4	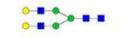	938.03	927.37	932.70
H3N5F1	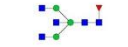	946.38	935.71	941.04
H4N5	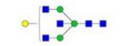	951.71		
H5N4F1	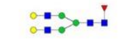	986.72	976.06	981.39
H4N5F1	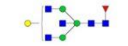	1000.39	989.73	995.06
H5N5	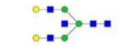	1005.73		1000.40
H4N4F1S1	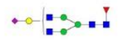	1029.73	1019.07	1024.40
H5N4S1	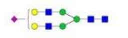	1035.06		
H6N4F1	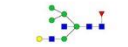		1030.08	
H5N5F1	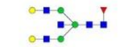	1054.41	1043.75	1049.08
H6N3F1S1	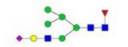		1059.41	
H5N4F1S1	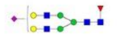	1083.75	1073.09	1078.42
H4N5F1S1	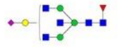	1097.43		1092.10
H5N5F1S1	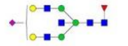	1151.44	1140.78	1146.11
H5N4F1S2	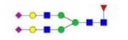	1180.78	1170.12	1175.45
H3N3F1	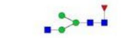	810.99	800.33	805.66
H4N3F1	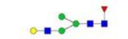	865.01	854.35	859.68
H4N4S1	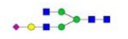	981.05		
H4N3F1S1	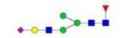		951.38	
H6N4S1	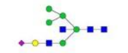			1083.75

H: hexose, N: N-acetylhexosamine, F: fucose. Green circle: mannose, yellow circle: galactose, blue square: N-acetylglucosamine, red triangle: fucose, pink diamond: N-acetylneuraminic acid.

Table S2. Subclass-specific glycosylation trait calculations. The individual glycoforms were grouped based on their glycosylation features as previously described for IgG glycopeptides in humans [37]. The depictions of the glycosylation traits show the minimally required composition to contribute to a trait. .

Glycosylation trait	Depiction	Description	Calculation
IgG1 Hybrids	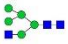	Fraction of hybrid glycans on IgG1	(IgGI_H3N3F1 + IgGI_H4N3F1)/ (IgGI_H3N4 + IgGI_H3N4F1 + IgGI_H4N4 + IgGI_H3N5 + IgGI_H4N4F1 + IgGI_H5N4 + IgGI_H3N5F1 + IgGI_H4N5 + IgGI_H4N4S1 + IgGI_H5N4F1 + IgGI_H4N5F1 + IgGI_H5N5 + IgGI_H4N4F1S1 + IgGI_H5N4S1 + IgGI_H5N5F1 + IgGI_H5N4F1S1 + IgGI_H4N5F1S1 + IgGI_H5N5F1S1 + IgGI_H5N4F1S2)
IgG1 Bisection	0 - 2x 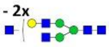	Bisection on IgG1	(IgGI_H3N5 + IgGI_H3N5F1 + IgGI_H4N5 + IgGI_H4N5F1 + IgGI_H5N5 + IgGI_H5N5F1 + IgGI_H4N5F1S1 + IgGI_H5N5F1S1)/ (IgGI_H3N4 + IgGI_H3N4F1 + IgGI_H4N4 + IgGI_H3N5 + IgGI_H4N4F1 + IgGI_H5N4 + IgGI_H3N5F1 + IgGI_H4N5 + IgGI_H4N4S1 + IgGI_H5N4F1 + IgGI_H4N5F1 + IgGI_H5N5 + IgGI_H4N4F1S1 + IgGI_H5N4S1 + IgGI_H5N5F1 + IgGI_H5N4F1S1 + IgGI_H4N5F1S1 + IgGI_H5N5F1S1 + IgGI_H5N4F1S2)
IgG1 Fucosylation	0 - 2x 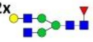	Fucosylation on IgG1	(IgGI1H3N4F1 + IgGI1H4N4F1 + IgGI1H3N5F1 + IgGI1H5N4F1 + IgGI1H4N5F1 + IgGI1H4N4F1S1 + IgGI1H5N5F1 + IgGI1H5N4F1S1 + IgGI1H4N5F1S1 + IgGI1H5N5F1S1 + IgGI1H5N4F1S2) / (IgGI_H3N4 + IgGI_H3N4F1 + IgGI_H4N4 + IgGI_H3N5 + IgGI_H4N4F1 + IgGI_H5N4 + IgGI_H3N5F1 + IgGI_H4N5 + IgGI_H4N4S1 + IgGI_H5N4F1 + IgGI_H4N5F1 + IgGI_H5N5 + IgGI_H4N4F1S1 + IgGI_H5N4S1 + IgGI_H5N5F1 + IgGI_H5N4F1S1 + IgGI_H4N5F1S1 + IgGI_H5N5F1S1 + IgGI_H5N4F1S2)
IgG1 Galactosylation	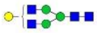	Galactosylation per antenna of diantennary glycans on IgG1	(1/2 *(IgGI_H4N4 + IgGI_H4N4F1 + IgGI_H4N5 + IgGI_H4N4S1 + IgGI_H4N5F1 + IgGI_H4N4F1S1 + IgGI_H4N5F1S1)+(IgGI_H5N4 + IgGI_H5N4F1 + IgGI_H5N5 + IgGI_H5N4S1 + IgGI_H5N5F1 + IgGI_H4N4S1 + IgGI_H5N5 + IgGI_H5N4S1)) / (IgGI_H3N4 + IgGI_H3N4F1 + IgGI_H4N4 + IgGI_H3N5 + IgGI_H4N4F1 + IgGI_H5N4 + IgGI_H3N5F1 + IgGI_H4N5 + IgGI_H4N4S1 + IgGI_H5N4F1 + IgGI_H4N5F1 + IgGI_H5N5 + IgGI_H4N4F1S1 + IgGI_H5N4S1 + IgGI_H5N5F1 + IgGI_H5N4F1S1 + IgGI_H4N5F1S1 + IgGI_H5N5F1S1 + IgGI_H5N4F1S2)
IgG1 Sialylation	0 - 2x 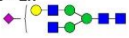	Sialylation per antenna of diantennary glycans on IgG1	(1/2 *(IgGI_H4N4S1 + IgGI_H4N4F1S1 + IgGI_H5N4S1 + IgGI_H5N4F1S1 + IgGI_H4N5F1S1 + IgGI_H5N5F1S1) + IgGI_H5N4F1S2 / (IgGI_H3N4 + IgGI_H3N4F1 + IgGI_H4N4 + IgGI_H3N5 + IgGI_H4N4F1 + IgGI_H5N4 + IgGI_H3N5F1 + IgGI_H4N5 + IgGI_H4N4S1 + IgGI_H5N4F1 + IgGI_H4N5F1 + IgGI_H5N5 + IgGI_H4N4F1S1 + IgGI_H5N4S1 + IgGI_H5N5F1 + IgGI_H5N4F1S1 + IgGI_H4N5F1S1 + IgGI_H5N5F1S1 + IgGI_H5N4F1S2)
IgG1 Galactosylation w/o Fucose	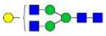	Galactosylation per antenna of diantennary glycans on IgG1 without fucose	(1/2 *(IgGI_H4N4 + IgGI_H4N5 + IgGI_H4N4S1) + (IgGI_H5N4 + IgGI_H5N5 + IgGI_H5N4S1) / (IgGI_H3N4 + IgGI_H4N4 + IgGI_H3N5 + IgGI_H5N4 + IgGI_H4N5 + IgGI_H4N4S1 + IgGI_H5N5 + IgGI_H5N4S1)
IgG1 Sialylation per galactose	0 - 2x 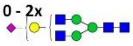	Sialylation per galactose of diantennary glycans on IgG1	IgG1 Sialylation / IgG1 Galactosylation
IgG2 Hybrids	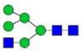	Fraction of hybrid glycans on IgG2	(IgGII_H3N3F1 + IgGII_H4N3F1 + IgGII_H4N3F1S1 + IgGII_H6N4F1 + IgGII_H6N3F1S1) / (IgGII1H3N4 + IgGII1H3N4F1 + IgGII1H4N4 + IgGII1H4N4F1 + IgGII1H5N4 + IgGII1H3N5F1 + IgGII1H5N4F1 + IgGII1H4N5F1 + IgGII1H4N4F1S1 + IgGII1H5N5F1 + IgGII1H5N4F1S1 + IgGII1H5N5F1S1 + IgGII1H5N4F1S2)
IgG2 Bisection	0 - 2x 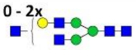	Bisection on IgG2	(IgGII_H3N5F1 + IgGII_H4N5F1 + IgGII_H5N5F1 + IgGII_H5N5F1S1) / (IgGII1H3N4 + IgGII1H3N4F1 + IgGII1H4N4 + IgGII1H4N4F1 + IgGII1H5N4 + IgGII1H3N5F1 + IgGII1H5N4F1 + IgGII1H4N5F1 + IgGII1H4N4F1S1 + IgGII1H5N5F1 + IgGII1H5N4F1S1 + IgGII1H5N5F1S1 + IgGII1H5N4F1S2)
IgG2 Fucosylation	0 - 2x 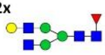	Fucosylation on IgG2	(IgGII_H3N4F1 + IgGII_H4N4F1 + IgGII_H3N5F1 + IgGII_H5N4F1 + IgGII_H4N5F1 + IgGII_H4N4F1S1 + IgGII_H5N5F1 + IgGII_H5N4F1S1 + IgGII_H5N5F1S1 + IgGII_H5N4F1S2) / (IgGII1H3N4 + IgGII1H3N4F1 + IgGII1H4N4 + IgGII1H4N4F1 + IgGII1H5N4 + IgGII1H3N5F1 + IgGII1H5N4F1 + IgGII1H4N5F1 + IgGII1H4N4F1S1 + IgGII1H5N5F1 + IgGII1H5N4F1S1 + IgGII1H5N5F1S1 + IgGII1H5N4F1S2)
IgG2 Galactosylation	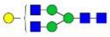	Galactosylation per antenna of diantennary glycans on IgG2	(1/2*(IgGII_H4N4 + IgGII_H4N4F1 + IgGII_H4N5F1 + IgGII_H4N4F1S1) + (IgGII_H5N4 + IgGII_H5N4F1 + IgGII_H5N5F1 + IgGII_H5N4F1S1 + IgGII_H5N5F1S1 + IgGII_H5N4F1S2)) / (IgGII1H3N4 + IgGII1H3N4F1 + IgGII1H4N4 + IgGII1H4N4F1 + IgGII1H5N4 + IgGII1H3N5F1 + IgGII1H5N4F1 + IgGII1H4N5F1 + IgGII1H4N4F1S1 + IgGII1H5N5F1 + IgGII1H5N4F1S1 + IgGII1H5N5F1S1 + IgGII1H5N4F1S2)
IgG2 Sialylation	0 - 2x 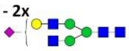	Sialylation per antenna of diantennary glycans on IgG2	(1/2*(IgGII_H4N4F1S1 + IgGII_H5N4F1S1 + IgGII_H5N5F1S1) + IgGII_H5N4F1S2) / (IgGII1H3N4 + IgGII1H3N4F1 + IgGII1H4N4 + IgGII1H4N4F1 + IgGII1H5N4 + IgGII1H3N5F1 + IgGII1H5N4F1 + IgGII1H4N5F1 + IgGII1H4N4F1S1 + IgGII1H5N5F1 + IgGII1H5N4F1S1 + IgGII1H5N5F1S1 + IgGII1H5N4F1S2)
IgG2 Galactosylation w/o Fucose	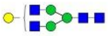	Galactosylation per antenna of diantennary glycans on IgG2 without fucose	(1/2* IgGII_H4N4 + IgGII_H5N4) / (IgGII_H3N4 + IgGII_H4N4 + IgGII_H5N4)
IgG2 Sialylation per galactose	0 - 2x 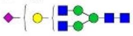	Sialylation per galactose of diantennary glycans on IgG2	IgGII Sialylation / IgGII Galactosylation
IgG4 Hybrids	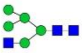	Fraction of hybrid glycans on IgG4	(IgGIV_H3N3F1 + IgGIV_H4N3F1 + IgGIV_H6N4S1)/ (IgGIV_H3N4F1 + IgGIV_H4N4 + IgGIV_H4N4F1 + IgGIV_H5N4 + IgGIV_H3N5F1 + IgGIV_H5N4F1 + IgGIV_H4N5F1 + IgGIV_H5N5 + IgGIV_H4N4F1S1 + IgGIV_H5N5F1 + IgGIV_H5N4F1S1 + IgGIV_H4N5F1S1 + IgGIV_H5N5F1S1 + IgGIV_H5N4F1S2)
IgG4 Bisection	0 - 2x 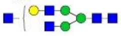	Bisection on IgG4	(IgGIV_H3N5F1 + IgGIV_H4N5F1 + IgGIV_H5N5 + IgGIV_H5N5F1 + IgGIV_H4N5F1S1 + IgGIV_H5N5F1S1)/ (IgGIV_H3N4F1 + IgGIV_H4N4 + IgGIV_H4N4F1 + IgGIV_H5N4 + IgGIV_H3N5F1 + IgGIV_H5N4F1 + IgGIV_H4N5F1 + IgGIV_H5N5 + IgGIV_H4N4F1S1 + IgGIV_H5N5F1 + IgGIV_H5N4F1S1 + IgGIV_H4N5F1S1 + IgGIV_H5N5F1S1 + IgGIV_H5N4F1S2)
IgG4 Fucosylation	0 - 2x 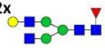	Fucosylation on IgG4	(IgGIV_H3N4F1 + IgGIV_H4N4F1 + IgGIV_H3N5F1 + IgGIV_H5N4F1 + IgGIV_H4N5F1 + IgGIV_H4N4F1S1 + IgGIV_H5N5F1 + IgGIV_H5N4F1S1 + IgGIV_H4N5F1S1 + IgGIV_H5N5F1S1 + IgGIV_H5N4F1S2)/ (IgGIV_H3N4F1 + IgGIV_H4N4 + IgGIV_H4N4F1 + IgGIV_H5N4 + IgGIV_H3N5F1 + IgGIV_H5N4F1 + IgGIV_H4N5F1 + IgGIV_H5N5 + IgGIV_H4N4F1S1 + IgGIV_H5N5F1 + IgGIV_H5N4F1S1 + IgGIV_H4N5F1S1 + IgGIV_H5N5F1S1 + IgGIV_H5N4F1S2)
IgG4 Galactosylation	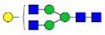	Galactosylation per antenna of diantennary glycans on IgG4	(1/2 * (IgGIV_H4N4 + IgGIV_H4N4F1 + IgGIV_H4N5F1 + IgGIV_H4N4F1S1 + IgGIV_H4N5F1S1) + (IgGIV_H5N4 + IgGIV_H5N4F1 + IgGIV_H5N5 + IgGIV_H5N5F1 + IgGIV_H5N4F1S1 + IgGIV_H5N5F1S1 + IgGIV_H5N4F1S2)) / (IgGIV_H3N4F1 + IgGIV_H4N4 + IgGIV_H4N4F1 + IgGIV_H5N4 + IgGIV_H3N5F1 + IgGIV_H5N4F1 + IgGIV_H4N5F1 + IgGIV_H5N5 + IgGIV_H4N4F1S1 + IgGIV_H5N5F1 + IgGIV_H5N4F1S1 + IgGIV_H4N5F1S1 + IgGIV_H5N5F1S1 + IgGIV_H5N4F1S2)
IgG4 Sialylation	0 - 2x 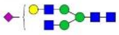	Sialylation per antenna of diantennary glycans on IgG4	(1/2 * (IgGIV_H4N4F1S1 + IgGIV_H5N4F1S1 + IgGIV_H4N5F1S1 + IgGIV_H5N5F1S1) + IgGIV_H5N4F1S2) / (IgGIV_H3N4F1 + IgGIV_H4N4 + IgGIV_H4N4F1 + IgGIV_H5N4 + IgGIV_H3N5F1 + IgGIV_H5N4F1 + IgGIV_H4N5F1 + IgGIV_H5N5 + IgGIV_H4N4F1S1 + IgGIV_H5N5F1 + IgGIV_H5N4F1S1 + IgGIV_H4N5F1S1 + IgGIV_H5N5F1S1 + IgGIV_H5N4F1S2)
IgG4 Galactosylation w/o Fucose	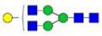	Galactosylation per antenna of diantennary glycans on IgG4 without fucose	(1/2 * IgGIV_H4N4 + (IgGIV_H5N4 + IgGIV_H5N5)) / (IgGIV_H4N4 + IgGIV_H5N4 + IgGIV_H5N5)
IgG4 Sialylation per galactose	0 - 2x 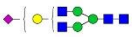	Sialylation per galactose of diantennary glycans on IgG4	IgGIV Sialylation / IgGIV Galactosylation

H: hexose, N: N-acetylhexosamine, F: fucose, S: *N*-acetylneuraminic acid. Green circle: mannose, yellow circle: galactose, blue square: *N*-acetylglucosamine, red triangle: fucose, pink diamond: *N*-acetylneuraminic acid.

Table S3. Clinical characteristics of the cohort. Age refers to years. High PLA2R was referred to patients showing an anti-PLA2R titre >999. Anti-PLA2R titre for PN patients is <10.

		N° (%) SUBJECTS /GROUP	average age	MIN AGE	MAX AGE	High PLA2R				Low PLA2R			
						N° SUBJECTS /GROUP	average age	MIN AGE	MAX AGE	N° SUBJECTS /GROUP	average age	MIN AGE	MAX AGE
**IMN -Idiopathic Membranous Nephropathy**	**All**	**57**	**58**	**28**	**85**	**29**	**60**	**28**	**85**	**28**	**58**	**37**	**78**
	**Females**	15 (26%)	62	40	83	6	65	41	83	9	60	40	74
	**Males**	42 (74%)	57	28	85	23	58	28	85	19	57	37	78
**PN -Pathological controls with not IMN Nephropathy**	**All**	**20**	**52**	**19**	**81**								
	**Females**	11 (55%)	52	19	81								
	**Males**	9 (45%)	53	35	73								
**CTRLS -healthy Controls**	**All**	**88**	**56**	**17**	**87**								
	**Females**	31 (35%)	56	17	86								
	**Males**	57 (65%)	56	28	87								

Table S4. Clinical characteristics of the pathological controls (n=20) affected by nephropathies different from IMN. Age refers to years.

DIAGNOSIS	N° subjects	N° Females	N° Males	Average Age	Min Age	Max Age
Amyloidosis nephropathy (AN)	2	1	1	57	35	78
Fabry disease (FD)	1	1	0	49	49	49
Focal glomerulosclerosis (FGS)	3	1	2	63	51	81
Focal Segmental glomerulosclerosis (FSGS)	2	2	0	61	49	72
Henoch-Schönlein purpura with proteinuria (HSP)	1	1	0	19	19	19
IgA nephropathy (IgAN)	2	0	2	68	65	71
Lupus Membranous Nephropathy (LMN)	2	1	1	30	23	37
Minimal Change Disease (MCD)	3	2	1	53	40	71
Minimal Change Disease / Focal Segmental glomerulosclerosis (MCD/FSGS)	1	1	0	40	40	40
Membranoproliferative glomerulonephritis (MPGN)	3	1	2	54	44	73

Table S5. Description of demographic characteristics and glyco-forms*, overall and by groups (IMN = Idiopathic Membranous Nephropathy; CTRL = Healthy controls; PN = Pathological controls with non-IMN nephropathy). Age refers to years. Results are reported as n (%) or median (Q1-Q3) as appropriate.

	Overall	CTRL	IMN	PN
Variables	n=165	n=88	n=57	n=20
Gender - F	57 (34.5)	31 (35.2)	15 (26.3)	11 (55.0)
M	108 (65.5)	57 (64.8)	42 (73.7)	9 (45.0)
Age	56.00 [43.00, 71.00]	56.00 [41.75, 70.25]	60.00 [44.00, 71.00]	49.00 [40.00, 71.00]
PLA2R - high	29 (50.9)	0	29 (50.9)	0
low	28 (49.1)	0	28 (49.1)	0
IgG1_hy	0.01 [0.00, 0.01]	0.01 [0.00, 0.01]	0.01 [0.01, 0.01]	0.01 [0.01, 0.01]
IgG1_fuc	0.92 [0.89, 0.94]	0.91 [0.89, 0.94]	0.92 [0.90, 0.94]	0.93 [0.89, 0.94]
IgG1_bisec	0.16 [0.14, 0.19]	0.17 [0.15, 0.19]	0.16 [0.13, 0.19]	0.16 [0.14, 0.18]
IgG1_gal	0.53 [0.47, 0.58]	0.56 [0.49, 0.61]	0.50 [0.44, 0.54]	0.51 [0.43, 0.56]
IgG1_gal_not_fucos	0.58 [0.52, 0.63]	0.59 [0.54, 0.65]	0.58 [0.52, 0.61]	0.57 [0.53, 0.64]
IgG1_sial	0.07 [0.06, 0.09]	0.08 [0.07, 0.10]	0.07 [0.06, 0.08]	0.07 [0.06, 0.09]
IgG1_sial.gal	0.14 [0.13, 0.16]	0.15 [0.14, 0.16]	0.14 [0.13, 0.15]	0.14 [0.13, 0.16]
IgG2_hy	0.01 [0.01, 0.01]	0.01 [0.01, 0.01]	0.01 [0.01, 0.01]	0.01 [0.01, 0.01]
IgG2_fuc	0.99 [0.99, 0.99]	0.99 [0.99, 0.99]	0.99 [0.99, 0.99]	0.99 [0.99, 0.99]
IgG2_bisec	0.13 [0.11, 0.14]	0.13 [0.12, 0.15]	0.12 [0.10, 0.14]	0.11 [0.10, 0.13]
IgG2_gal	0.44 [0.38, 0.50]	0.45 [0.40, 0.52]	0.42 [0.38, 0.47]	0.40 [0.36, 0.51]
IgG2_gal_not_fucos	0.47 [0.45, 0.49]	0.47 [0.44, 0.49]	0.47 [0.45, 0.50]	0.47 [0.45, 0.48]
IgG2_sial	0.07 [0.06, 0.09]	0.07 [0.06, 0.09]	0.07 [0.06, 0.08]	0.07 [0.06, 0.09]
IgG2_sial.gal	0.17 [0.16, 0.18]	0.17 [0.16, 0.18]	0.17 [0.16, 0.18]	0.17 [0.16, 0.19]
IgG4_hy	0.02 [0.01, 0.03]	0.02 [0.02, 0.03]	0.01 [0.01, 0.02]	0.02 [0.01, 0.04]
IgG4_fuc	0.91 [0.83, 0.94]	0.89 [0.82, 0.93]	0.93 [0.89, 0.95]	0.88 [0.79, 0.94]
IgG4_bisec	0.19 [0.16, 0.23]	0.19 [0.16, 0.22]	0.18 [0.15, 0.23]	0.17 [0.15, 0.23]
IgG4_gal	0.50 [0.44, 0.56]	0.54 [0.48, 0.60]	0.45 [0.38, 0.50]	0.51 [0.43, 0.57]
IgG4_gal_not_fucos	0.83 [0.80, 0.85]	0.84 [0.81, 0.86]	0.80 [0.78, 0.83]	0.81 [0.79, 0.85]
IgG4_sial	0.08 [0.07, 0.11]	0.09 [0.07, 0.11]	0.08 [0.07, 0.10]	0.09 [0.05, 0.10]
IgG4_sial.gal	0.18 [0.15, 0.20]	0.17 [0.15, 0.20]	0.19 [0.17, 0.21]	0.17 [0.13, 0.21]

*hy = Hybrid glycoforms; fuc = with Fucosylation; bisec = with Bisection; gal =with Galactosylation; sial = with Sialylation; gal_not fucos = Galactosylation of non-fucosylated glycoforms; sial_ gal = Sialylation per Galactose.

Table S6. Number of subjects classified for age and gender in each group.

	BM	OM	BF	OF	Total
CTRL	34	23	16	15	88
IMN	22	20	6	9	57
PN	6	3	7	4	20
Total	62	46	29	28	165

BM (Below60y Males)= Age<60 Gender = Male
OM (Over 60y Males)= Age>=60 Gender = Male
BF (Below60y Females)= Age<60 Gender = Female
OF ((Below60y Females)= Age>=60 Gender = Female

Table S7. Average and IQR related to the normalised abundances of the seven glycosylation traits* for IgG1 (a), IgG2(b), IgG4 in IMN. The dataset of subjects was divided in categories based on sex (F= Female, M= Males) and age (O=Over 60 years old -age>=60; B= below 60 years old- age<60) according to Figure S2. The corresponding p-value (p) and number of subjects are shown.

	Overall	BF	BM	OF	OM	
**n**	57	6	22	9	20	**p**
**IgG1_hy**	0.01 [0.01, 0.01]	0.01 [0.00, 0.01]	0.01 [0.01, 0.01]	0.01 [0.01, 0.01]	0.01 [0.01, 0.01]	0.303
**IgG1_fuc**	0.92 [0.90, 0.94]	0.91 [0.90, 0.94]	0.91 [0.90, 0.94]	0.90 [0.86, 0.94]	0.92 [0.91, 0.94]	0.689
**IgG1_bisec**	0.16 [0.13, 0.19]	0.13 [0.12, 0.14]	0.15 [0.13, 0.18]	0.20 [0.15, 0.21]	0.17 [0.15, 0.18]	0.046
**IgG1_gal**	0.50 [0.44, 0.54]	0.54 [0.53, 0.60]	0.53 [0.45, 0.56]	0.42 [0.38, 0.49]	0.47 [0.45, 0.51]	0.003
**IgG1_gal_not_fucos**	0.58 [0.52, 0.61]	0.61 [0.59, 0.64]	0.59 [0.54, 0.62]	0.49 [0.45, 0.53]	0.58 [0.52, 0.60]	0.005
**IgG1_sial**	0.07 [0.06, 0.08]	0.08 [0.07, 0.09]	0.07 [0.06, 0.08]	0.06 [0.05, 0.07]	0.07 [0.06, 0.08]	0.029
**IgG1_sial.gal**	0.14 [0.13, 0.15]	0.15 [0.14, 0.15]	0.14 [0.13, 0.15]	0.14 [0.12, 0.15]	0.14 [0.14, 0.15]	0.508
**IgG2_hy**	0.01 [0.01, 0.01]	0.01 [0.01, 0.01]	0.01 [0.01, 0.01]	0.01 [0.01, 0.01]	0.01 [0.01, 0.01]	0.02
**IgG2_fuc**	0.99 [0.99, 0.99]	0.99 [0.99, 0.99]	0.99 [0.99, 0.99]	0.98 [0.98, 0.99]	0.99 [0.99, 0.99]	0.343
**IgG2_bisec**	0.12 [0.10, 0.14]	0.11 [0.09, 0.12]	0.12 [0.10, 0.13]	0.14 [0.12, 0.15]	0.12 [0.10, 0.14]	0.286
**IgG2_gal**	0.42 [0.38, 0.47]	0.47 [0.47, 0.51]	0.43 [0.41, 0.48]	0.35 [0.28, 0.38]	0.41 [0.35, 0.45]	<0.001
**IgG2_gal_not_fucos**	0.47 [0.45, 0.50]	0.47 [0.47, 0.48]	0.48 [0.45, 0.50]	0.43 [0.42, 0.46]	0.47 [0.46, 0.49]	0.006
**IgG2_sial**	0.07 [0.06, 0.08]	0.08 [0.08, 0.09]	0.07 [0.07, 0.09]	0.06 [0.05, 0.06]	0.07 [0.06, 0.08]	0.001
**IgG2_sial.gal**	0.17 [0.16, 0.18]	0.17 [0.17, 0.18]	0.17 [0.16, 0.18]	0.17 [0.16, 0.18]	0.18 [0.16, 0.19]	0.416
**IgG4_hy**	0.01 [0.01, 0.02]	0.01 [0.01, 0.02]	0.01 [0.01, 0.02]	0.01 [0.01, 0.02]	0.01 [0.01, 0.02]	0.898
**IgG4_fuc**	0.93 [0.89, 0.95]	0.93 [0.92, 0.96]	0.93 [0.89, 0.95]	0.92 [0.90, 0.94]	0.93 [0.90, 0.96]	0.884
**IgG4_bisec**	0.18 [0.15, 0.23]	0.16 [0.16, 0.19]	0.17 [0.13, 0.21]	0.23 [0.19, 0.25]	0.20 [0.17, 0.23]	0.178
**IgG4_gal**	0.45 [0.38, 0.50]	0.51 [0.50, 0.56]	0.46 [0.42, 0.50]	0.42 [0.33, 0.46]	0.41 [0.35, 0.46]	0.003
**IgG4_gal_not_fucos**	0.80 [0.78, 0.83]	0.83 [0.80, 0.84]	0.82 [0.80, 0.84]	0.78 [0.77, 0.80]	0.80 [0.78, 0.82]	0.047
**IgG4_sial**	0.08 [0.07, 0.10]	0.11 [0.11, 0.12]	0.08 [0.08, 0.10]	0.08 [0.05, 0.09]	0.07 [0.06, 0.09]	0.01
**IgG4_sial.gal**	0.19 [0.17, 0.21]	0.21 [0.21, 0.22]	0.19 [0.17, 0.20]	0.19 [0.17, 0.20]	0.18 [0.18, 0.20]	0.368

*hy=Hybrid glycoforms; fuc= with Fucosylation; bisec= with Bisection; gal=with Galactosylation; sial=with Sialylation; gal_not fucos= Galactosylation of non-fucosylated glycoforms; sial_ gal= Sialylation per Galactose.

Table S8. Average and IQR related to the normalised abundances of the seven glycosylation traits for IgG1 (a), IgG2(b), IgG4 in CTRLs matched with IMN. The dataset of subjects was divided in categories based on sex (F= Female, M= Males) and age (O=Over 60 years old -age>=60; B= below 60 years old- age<60) according to Figure S2. The corresponding p-value (p) and number of subjects are shown.

	Overall	BF	BM	OF	OM	
**n**	84	12	34	15	23	**p**
**IgG1_hy**	0.00 [0.00, 0.01]	0.00 [0.00, 0.00]	0.01 [0.00, 0.01]	0.01 [0.00, 0.01]	0.01 [0.00, 0.01]	<0.001
**IgG1_fuc**	0.91 [0.89, 0.93]	0.92 [0.86, 0.93]	0.92 [0.90, 0.94]	0.91 [0.90, 0.93]	0.90 [0.88, 0.93]	0.567
**IgG1_bisec**	0.17 [0.15, 0.19]	0.16 [0.15, 0.17]	0.16 [0.14, 0.18]	0.20 [0.18, 0.22]	0.18 [0.16, 0.20]	0.001
**IgG1_gal**	0.56 [0.49, 0.61]	0.63 [0.62, 0.65]	0.59 [0.53, 0.61]	0.49 [0.46, 0.52]	0.48 [0.44, 0.54]	<0.001
**IgG1_gal_not_fucos**	0.59 [0.53, 0.65]	0.69 [0.67, 0.70]	0.62 [0.58, 0.65]	0.51 [0.49, 0.56]	0.56 [0.49, 0.58]	<0.001
**IgG1_sial**	0.08 [0.07, 0.10]	0.10 [0.09, 0.11]	0.09 [0.08, 0.10]	0.07 [0.06, 0.08]	0.07 [0.06, 0.08]	<0.001
**IgG1_sial.gal**	0.15 [0.13, 0.16]	0.17 [0.14, 0.17]	0.15 [0.14, 0.16]	0.14 [0.13, 0.14]	0.14 [0.13, 0.15]	0.012
**IgG2_hy**	0.01 [0.01, 0.01]	0.01 [0.01, 0.01]	0.01 [0.01, 0.01]	0.01 [0.01, 0.01]	0.01 [0.01, 0.01]	<0.001
**IgG2_fuc**	0.99 [0.99, 0.99]	0.99 [0.99, 0.99]	0.99 [0.99, 0.99]	0.99 [0.99, 0.99]	0.99 [0.99, 0.99]	0.991
**IgG2_bisec**	0.13 [0.12, 0.15]	0.14 [0.13, 0.14]	0.13 [0.11, 0.14]	0.16 [0.14, 0.17]	0.14 [0.12, 0.15]	0.004
**IgG2_gal**	0.44 [0.39, 0.51]	0.56 [0.51, 0.56]	0.47 [0.44, 0.52]	0.39 [0.35, 0.42]	0.40 [0.38, 0.45]	<0.001
**IgG2_gal_not_fucos**	0.47 [0.44, 0.49]	0.49 [0.48, 0.50]	0.48 [0.45, 0.49]	0.44 [0.43, 0.45]	0.45 [0.43, 0.49]	<0.001
**IgG2_sial**	0.07 [0.06, 0.09]	0.10 [0.09, 0.10]	0.08 [0.07, 0.10]	0.06 [0.05, 0.07]	0.07 [0.06, 0.08]	<0.001
**IgG2_sial.gal**	0.17 [0.16, 0.18]	0.18 [0.17, 0.19]	0.17 [0.16, 0.19]	0.16 [0.15, 0.17]	0.17 [0.15, 0.18]	0.054
**IgG4_hy**	0.02 [0.02, 0.03]	0.03 [0.03, 0.04]	0.02 [0.01, 0.03]	0.02 [0.02, 0.03]	0.02 [0.02, 0.03]	0.041
**IgG4_fuc**	0.89 [0.82, 0.93]	0.89 [0.83, 0.90]	0.92 [0.82, 0.94]	0.86 [0.77, 0.90]	0.85 [0.83, 0.90]	0.047
**IgG4_bisec**	0.19 [0.17, 0.23]	0.20 [0.19, 0.21]	0.17 [0.15, 0.20]	0.24 [0.20, 0.26]	0.20 [0.18, 0.22]	0.003
**IgG4_gal**	0.53 [0.48, 0.59]	0.60 [0.55, 0.63]	0.56 [0.53, 0.61]	0.49 [0.43, 0.52]	0.48 [0.42, 0.52]	<0.001
**IgG4_gal_not_fucos**	0.84 [0.81, 0.86]	0.87 [0.87, 0.88]	0.85 [0.83, 0.87]	0.83 [0.80, 0.84]	0.80 [0.79, 0.84]	<0.001
**IgG4_sial**	0.09 [0.07, 0.11]	0.10 [0.09, 0.11]	0.10 [0.08, 0.12]	0.08 [0.07, 0.09]	0.08 [0.06, 0.08]	<0.001
**IgG4_sial.gal**	0.17 [0.15, 0.20]	0.19 [0.16, 0.20]	0.19 [0.15, 0.21]	0.16 [0.14, 0.18]	0.16 [0.15, 0.18]	0.072

*hy=Hybrid glycoforms; fuc= with Fucosylation; bisec= with Bisection; gal=with Galactosylation; sial=with Sialylation; gal_not fucos= Galactosylation of non-fucosylated glycoforms; sial_ gal= Sialylation per Galactose

Table S9. Average and IQR related to the normalised abundances of the seven glycosylation traits* for IgG1 (a), IgG2(b), IgG4 in PN. The dataset of subjects was divided in categories based on sex (F= Female, M= Males) and age (O=Over 60 years old -age>=60; B= below 60 years old- age<60) according to Figure S2. The corresponding p-value (p) and number of subjects are shown.

	Overall	BF	BM	OF	OM	
n	20	7	6	4	3	p
IgG1_hy	0.01 [0.00, 0.01]	0.00 [0.00, 0.01]	0.01 [0.01, 0.01]	0.01 [0.01, 0.01]	0.01 [0.01, 0.01]	0.015
IgG1_fuc	0.93 [0.89, 0.94]	0.94 [0.91, 0.94]	0.93 [0.91, 0.94]	0.93 [0.87, 0.93]	0.88 [0.88, 0.92]	0.809
IgG1_bisec	0.16 [0.14, 0.18]	0.16 [0.16, 0.18]	0.15 [0.15, 0.16]	0.17 [0.14, 0.21]	0.14 [0.13, 0.14]	0.258
IgG1_gal	0.51 [0.43, 0.56]	0.57 [0.52, 0.63]	0.46 [0.40, 0.53]	0.39 [0.35, 0.44]	0.56 [0.52, 0.57]	0.019
IgG1_gal_not_fucos	0.57 [0.53, 0.64]	0.64 [0.59, 0.69]	0.57 [0.51, 0.59]	0.50 [0.47, 0.53]	0.57 [0.55, 0.62]	0.072
IgG1_sial	0.07 [0.06, 0.09]	0.07 [0.07, 0.10]	0.06 [0.05, 0.08]	0.06 [0.05, 0.06]	0.09 [0.08, 0.09]	0.116
IgG1_sial.gal	0.14 [0.13, 0.16]	0.14 [0.13, 0.15]	0.14 [0.12, 0.14]	0.15 [0.14, 0.15]	0.16 [0.15, 0.16]	0.315
IgG2_hy	0.01 [0.01, 0.01]	0.01 [0.01, 0.01]	0.01 [0.01, 0.02]	0.01 [0.01, 0.01]	0.01 [0.01, 0.01]	0.018
IgG2_fuc	0.99 [0.99, 0.99]	0.99 [0.99, 0.99]	0.99 [0.99, 0.99]	0.98 [0.98, 0.99]	0.99 [0.99, 0.99]	0.616
IgG2_bisec	0.11 [0.10, 0.13]	0.13 [0.12, 0.14]	0.11 [0.10, 0.11]	0.14 [0.13, 0.15]	0.10 [0.10, 0.11]	0.045
IgG2_gal	0.40 [0.36, 0.51]	0.52 [0.43, 0.53]	0.38 [0.30, 0.47]	0.32 [0.29, 0.34]	0.44 [0.40, 0.47]	0.034
IgG2_gal_not_fucos	0.47 [0.45, 0.48]	0.48 [0.46, 0.48]	0.46 [0.44, 0.47]	0.45 [0.42, 0.48]	0.49 [0.48, 0.50]	0.295
IgG2_sial	0.07 [0.06, 0.09]	0.09 [0.06, 0.10]	0.06 [0.05, 0.08]	0.06 [0.05, 0.06]	0.07 [0.07, 0.09]	0.26
IgG2_sial.gal	0.17 [0.16, 0.19]	0.17 [0.15, 0.18]	0.17 [0.17, 0.18]	0.18 [0.16, 0.19]	0.17 [0.17, 0.18]	0.799
IgG4_hy	0.02 [0.01, 0.04]	0.03 [0.02, 0.05]	0.01 [0.01, 0.04]	0.01 [0.01, 0.02]	0.02 [0.02, 0.02]	0.484
IgG4_fuc	0.88 [0.79, 0.94]	0.80 [0.78, 0.94]	0.89 [0.72, 0.94]	0.88 [0.80, 0.92]	0.93 [0.88, 0.94]	0.928
IgG4_bisec	0.17 [0.15, 0.23]	0.23 [0.18, 0.25]	0.17 [0.14, 0.19]	0.19 [0.15, 0.25]	0.16 [0.16, 0.17]	0.311
IgG4_gal	0.51 [0.43, 0.57]	0.56 [0.50, 0.65]	0.48 [0.35, 0.58]	0.38 [0.27, 0.50]	0.51 [0.46, 0.52]	0.211
IgG4_gal_not_fucos	0.81 [0.79, 0.85]	0.86 [0.85, 0.87]	0.79 [0.75, 0.83]	0.76 [0.71, 0.80]	0.81 [0.81, 0.83]	0.013
IgG4_sial	0.09 [0.05, 0.10]	0.10 [0.08, 0.11]	0.07 [0.05, 0.09]	0.06 [0.05, 0.08]	0.10 [0.08, 0.11]	0.426
IgG4_sial.gal	0.17 [0.13, 0.21]	0.18 [0.15, 0.21]	0.15 [0.14, 0.19]	0.16 [0.12, 0.20]	0.20 [0.17, 0.21]	0.721

*hy=Hybrid glycoforms; fuc= with Fucosylation; bisec= with Bisection; gal=with Galactosylation; sial=with Sialylation; gal_not fucos= Galactosylation of non-fucosylated glycoforms; sial_ gal= Sialylation per Galactose

Table S10. Average and IQR related to the normalised abundances of the seven glycosylation traits* for for IgG1 (a), IgG2(b), IgG4 in in CTRLs matched with PN. The dataset of subjects was divided in categories based on sex (F= Female, M= Males) and age (O=Over 60 years old -age>=60; B= below 60 years old- age<60) according to Figure S2. The corresponding p-value (p) and number of subjects are shown.

	Overall	BF	BM	OF	OM	
n	69	15	29	9	16	p
IgG1_hy	0.00 [0.00, 0.01]	0.00 [0.00, 0.00]	0.01 [0.00, 0.01]	0.01 [0.00, 0.01]	0.01 [0.00, 0.01]	0.006
IgG1_fuc	0.92 [0.89, 0.94]	0.93 [0.88, 0.95]	0.92 [0.89, 0.94]	0.91 [0.91, 0.93]	0.90 [0.87, 0.93]	0.555
IgG1_bisec	0.17 [0.15, 0.19]	0.15 [0.13, 0.16]	0.16 [0.14, 0.18]	0.20 [0.18, 0.22]	0.19 [0.17, 0.21]	<0.001
IgG1_gal	0.56 [0.50, 0.62]	0.64 [0.62, 0.66]	0.58 [0.52, 0.61]	0.48 [0.44, 0.53]	0.49 [0.46, 0.55]	<0.001
IgG1_gal_not_fucos	0.59 [0.54, 0.66]	0.70 [0.68, 0.72]	0.62 [0.58, 0.64]	0.51 [0.49, 0.53]	0.56 [0.49, 0.58]	<0.001
IgG1_sial	0.08 [0.07, 0.10]	0.10 [0.09, 0.11]	0.09 [0.08, 0.10]	0.06 [0.06, 0.08]	0.07 [0.06, 0.08]	<0.001
IgG1_sial.gal	0.15 [0.13, 0.16]	0.17 [0.14, 0.17]	0.15 [0.14, 0.16]	0.13 [0.13, 0.14]	0.15 [0.13, 0.15]	0.01
IgG2_hy	0.01 [0.01, 0.01]	0.01 [0.01, 0.01]	0.01 [0.01, 0.01]	0.01 [0.01, 0.01]	0.01 [0.01, 0.01]	<0.001
IgG2_fuc	0.99 [0.99, 0.99]	0.99 [0.99, 0.99]	0.99 [0.99, 0.99]	0.99 [0.99, 0.99]	0.99 [0.99, 0.99]	0.832
IgG2_bisec	0.13 [0.12, 0.15]	0.13 [0.11, 0.14]	0.13 [0.11, 0.13]	0.15 [0.14, 0.17]	0.14 [0.13, 0.17]	0.019
IgG2_gal	0.45 [0.40, 0.53]	0.56 [0.53, 0.56]	0.45 [0.43, 0.52]	0.38 [0.33, 0.39]	0.43 [0.39, 0.45]	<0.001
IgG2_gal_not_fucos	0.47 [0.45, 0.49]	0.50 [0.49, 0.51]	0.47 [0.45, 0.48]	0.44 [0.44, 0.44]	0.45 [0.43, 0.49]	<0.001
IgG2_sial	0.07 [0.06, 0.09]	0.10 [0.08, 0.11]	0.08 [0.06, 0.10]	0.06 [0.05, 0.06]	0.07 [0.07, 0.07]	<0.001
IgG2_sial.gal	0.17 [0.15, 0.18]	0.18 [0.16, 0.19]	0.17 [0.16, 0.19]	0.16 [0.15, 0.16]	0.17 [0.15, 0.18]	0.078
IgG4_hy	0.02 [0.02, 0.03]	0.03 [0.03, 0.03]	0.02 [0.01, 0.03]	0.02 [0.01, 0.02]	0.02 [0.02, 0.03]	0.011
IgG4_fuc	0.89 [0.83, 0.93]	0.89 [0.86, 0.92]	0.91 [0.82, 0.94]	0.89 [0.86, 0.90]	0.87 [0.82, 0.92]	0.399
IgG4_bisec	0.19 [0.17, 0.23]	0.19 [0.15, 0.20]	0.17 [0.15, 0.20]	0.23 [0.20, 0.26]	0.20 [0.19, 0.25]	0.015
IgG4_gal	0.54 [0.48, 0.60]	0.61 [0.57, 0.65]	0.56 [0.52, 0.60]	0.46 [0.42, 0.49]	0.48 [0.41, 0.51]	<0.001
IgG4_gal_not_fucos	0.85 [0.82, 0.87]	0.87 [0.86, 0.89]	0.85 [0.82, 0.86]	0.83 [0.80, 0.84]	0.83 [0.79, 0.84]	<0.001
IgG4_sial	0.09 [0.07, 0.11]	0.11 [0.10, 0.13]	0.10 [0.08, 0.12]	0.08 [0.07, 0.09]	0.08 [0.06, 0.08]	0.001
IgG4_sial.gal	0.17 [0.15, 0.20]	0.19 [0.17, 0.20]	0.18 [0.15, 0.21]	0.17 [0.15, 0.19]	0.16 [0.15, 0.17]	0.142

*hy=Hybrid glycoforms; fuc= with Fucosylation; bisec= with Bisection; gal=with Galactosylation; sial=with Sialylation; gal_not fucos= Galactosylation of non-fucosylated glycoforms; sial_ gal= Sialylation per Galactose
